# Resistance Gene Pyramiding in Wheat (*Triticum aestivum* L.): From Marker-Assisted Selection to Functional Validation

**DOI:** 10.3390/ijms27219918

**Published:** 2026-11-05

**Authors:** Michał T. Kwiatek, Roksana Bobrowska, Aleksandra Noweiska, Julia Spychała, Sylwia Mikołajczyk, Agnieszka Tomkowiak

**Affiliations:** 1 Plant Breeding and Acclimatization Institute—National Research Institute in Radzików, Radzików, 05-870 Błonie, Poland; a.noweiska@ihar.edu.pl (A.N.); j.spychala@ihar.edu.pl (J.S.); 2 Department of Genetics and Plant Breeding, Faculty of Agriculture, Horticulture and Biotechnology, Poznań University of Life Sciences, Dojazd 11, 60-632 Poznań, Poland; roksana.bobrowska@up.poznan.pl (R.B.); sylwia.mikolajczyk@up.poznan.pl (S.M.); agnieszka.tomkowiak@up.poznan.pl (A.T.)

**Keywords:** gene pyramiding, resistance, marker-assisted selection, genetic background, gene expression, genome editing

## Abstract

Gene pyramiding is widely used in wheat breeding to improve disease resistance; however, combining multiple resistance alleles does not necessarily provide stronger protection or long-term durability. Molecular markers, gene cloning and genome editing have greatly facilitated the tracking and pyramiding of resistance alleles. These approaches define the allelic composition of a line rather than the level of resistance it will express. This review examines why the effectiveness of resistance gene combinations remains difficult to predict despite advances in molecular tools. Evidence from leaf rust, stripe rust, stem rust, powdery mildew, eyespot and Fusarium head blight shows that the observed effects of combined resistance loci depend on the genetic background, gene interactions and environmental conditions, as well as on the methods used to assess disease development. We discuss how genetic characterization, expression analysis, functional assays and multi-environment phenotyping can provide complementary evidence for evaluating resistance gene combinations and how pathogen evolution and deployment strategies affect their durability. As genome editing expands the possibilities for combining resistance alleles, understanding their functional compatibility and their performance across genetic backgrounds remains essential for developing effective and durable resistance in wheat. The central conclusion of this review is that no marker profile, and no edited allele, is by itself evidence that a cultivar will be resistant in the field. Markers and gene editing shorten the route to a combination, but the resistance of that combination still must be validated phenotypically in the target backgrounds and environments.

## 1. Introduction

Wheat breeders have long combined resistance genes in a single genotype to improve disease resistance and reduce the risk of resistance breakdown. This strategy, known as gene pyramiding, rests on the assumption that a pathogen population is less likely to acquire virulence to several resistance genes simultaneously than to a single gene [1,2]. This is important because race-specific genes, once widely deployed, have repeatedly been overcome by new virulent pathotypes [3]. However, the presence of several resistance alleles does not necessarily translate into stronger or more durable resistance. The effectiveness of gene pyramiding depends not only on which genes are combined, but also on how they interact, on the genetic background and on the pathogen population. For most combinations of leaf rust resistance genes, disease severity is similar to that conferred by the most effective gene in the combination, whereas certain genes enhance the resistance conferred by their partners [4]. Moreover, the same gene combination can result in different levels of resistance among sister lines, which points to an additional effect of the genetic background [4]. Understanding these relationships remains a major challenge in resistance breeding [1,5].

Advances in molecular markers, sequencing and gene cloning have improved our ability to identify and combine resistance loci and have enabled the development of gene-specific markers [6]. Cloned genes can be combined directly as transgenes. For example, a cassette of five stem rust resistance genes introduced into wheat as a single locus conferred very high levels of field resistance, although only four of the five genes were shown to be functional, and a pathogen isolate virulent to several genes in the stack was subsequently identified [7]. Genome editing provides a further route, as illustrated by the simultaneous mutation of all three *Mlo* homoeologues in hexaploid wheat, which conferred heritable, broad-spectrum resistance to powdery mildew [8]. Nevertheless, confirming that specific resistance alleles are present does not reveal how they will perform in combination. Markers that are only linked to a resistance locus may also produce false-positive or false-negative results. For *Lr46* (*Lr46/Yr29*), four commonly used markers gave largely discordant results when applied to the same set of cultivars [9]. A marker-confirmed combination may confer effective resistance in one genetic background but behave differently in another. Introgressed resistance loci may also carry linked chromosomal segments that reduce yield or quality (linkage drag), so a resistant genotype is not necessarily agronomically acceptable [10]. Similarly, resistance observed under controlled conditions is not evidence of long-term durability. Durability can only be established retrospectively, after a resistance has remained effective during prolonged and widespread cultivation in environments favourable to the disease [11]. These limitations highlight the need to distinguish between the genetic identification of resistance genes and the experimental validation of resistance, which is the evidence breeders ultimately require before a line is advanced towards registration and commercial release.

Adult-plant resistance (APR) genes provide a useful model for examining these relationships. Unlike race-specific R genes, which are typically effective at all growth stages, APR genes usually function in adult plants and confer only partial resistance that still allows considerable disease development [5]. In wheat, *Lr34 (Lr34/Yr18/Sr57/Pm38/Ltn1)*, *Lr46* and *Lr67*, among other genes, are associated with partial and potentially durable resistance to several pathogens, including leaf rust, stripe rust, stem rust and powdery mildew [4,9]. The strength of the molecular evidence for their functions, however, varies considerably. *Lr34* encodes an ABC transporter [12] that transports abscisic acid. Notably, both the resistant and the susceptible *Lr34* variants transport this phytohormone, but only the resistant protein has been detected *in planta*, which suggests post-transcriptional regulation [13]. The resistance allele of *Lr67* encodes a variant hexose transporter that reduces glucose uptake through a dominant-negative interaction with functional transporters [14]. It has more recently been associated with anion fluxes when expressed in heterologous systems [15]. Thus, although both genes have been isolated and functionally validated, their mechanisms are still being refined. In contrast, the sequence of the gene underlying *Lr46* has not been published, and only candidate genes have been proposed [9]. Expression analyses of miRNAs associated with *Lr46* suggest their involvement in the resistance response [16], but such data provide indirect rather than causal evidence. These differences show that neither marker profiles nor transcript levels alone can establish how a resistance gene functions. The effects of APR genes may also depend on interactions with other resistance loci and on the genetic background of the recipient line [4]. When present alone, APR genes do not provide adequate protection under high disease pressure, but combinations of four or five such genes usually result in near-immunity [17]. In addition, *Lr34* induces a senescence-like phenotype at the tips of flag leaves, known as leaf tip necrosis [12]. Its strong, constitutive expression in transgenic barley severely reduced plant growth, whereas pathogen-inducible expression maintained resistance without this penalty [18]. This example illustrates that, in transgenic material, resistance effects must be distinguished from the effects of transgene expression level.

Studies of resistance to other wheat diseases, particularly eyespot and Fusarium head blight, further underline the importance of genetic background and environmental conditions, as well as the need to consider agronomic performance. For eyespot, the highly effective gene *Pch1* was introgressed from *Aegilops ventricosa* as part of a large chromosomal segment, and its use in European cultivars has long been limited by an associated yield penalty [10,19]. More recent mapping showed, however, that this yield penalty is genetically distinct from *Pch1* and environmentally unstable, so resistance and reduced yield can be separated by recombination [20]. For Fusarium head blight, near-isogenic lines developed from 13 breeding populations confirmed an average beneficial effect of the major QTL *Fhb1*. Yet a significant reduction in disease spread was observed in fewer than half of the 19 line pairs tested [21]. Together, these examples show that the value of a resistance gene combination can only be assessed within a specific genetic and environmental context.

**Figure 1:**
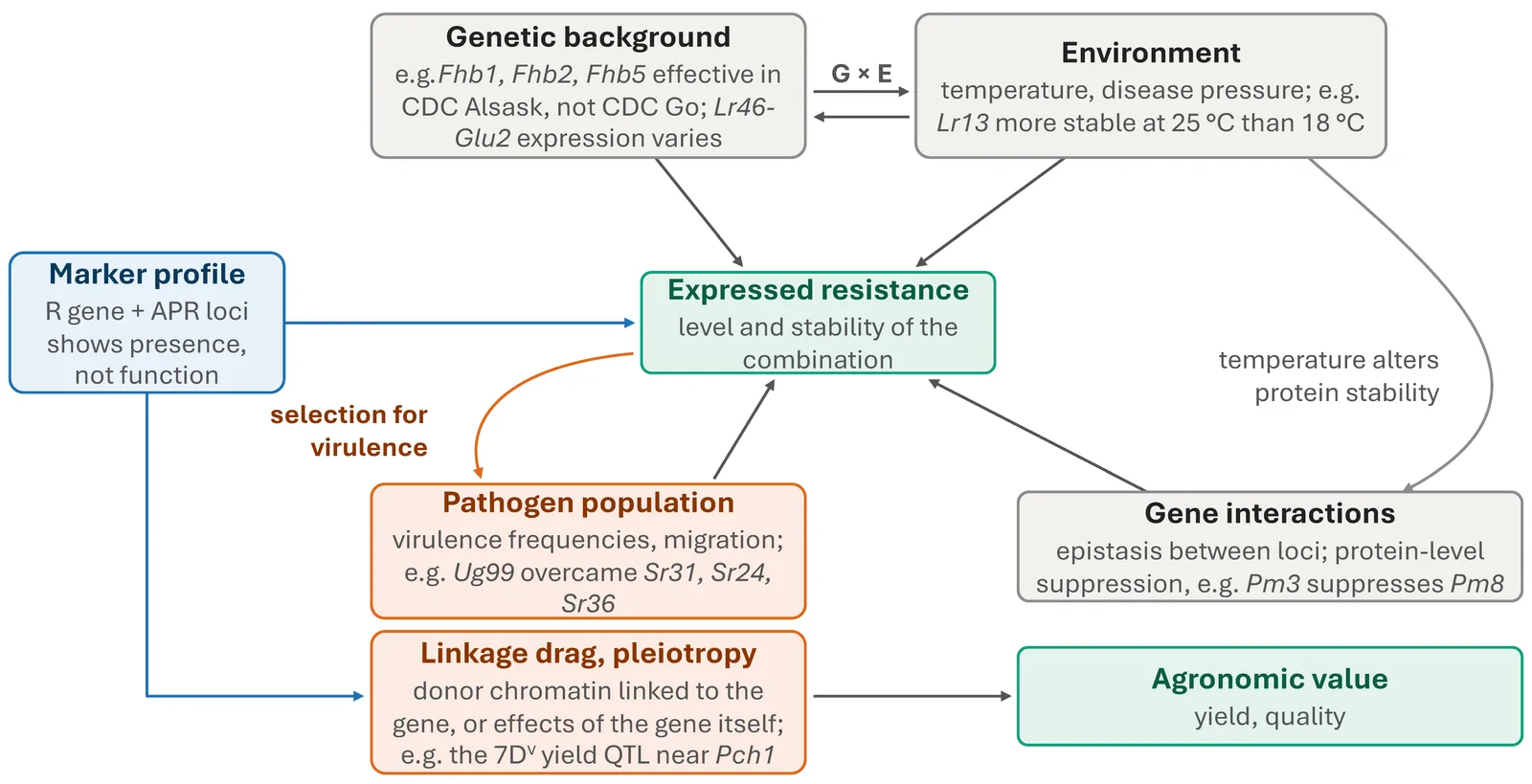
Factors affecting the performance of marker-defined resistance gene combinations in wheat. Molecular markers confirm the presence of resistance alleles, for example, a major race-specific (R) gene combined with adult-plant resistance (APR) loci. The level and stability of the resulting resistance depend on interactions between the combined genes, regulation at the protein level, the genetic background of the recipient line, the environment and the pathogen population. These factors are not independent (curved arrows): the effect of the genetic background depends on the environment (G × E), temperature alters the stability of some resistance proteins, the genetic background influences the expression of resistance-associated genes, and the deployment of resistance selects for virulence in the pathogen population, which in turn changes the resistance expressed. Examples are discussed in Section 2, Section 3, Section 4 and Section 5. The agronomic value of the combination may be reduced by linkage drag (effects of donor chromatin linked to the introgressed resistance locus) or by pleiotropic effects of the resistance genes themselves.

Previous reviews have addressed the theory of gene pyramiding and the durability of resistance [1,3,11] and the history and prospects of breeding for rust resistance [5]. Less attention has been paid to the gap between confirming that several resistance alleles are present in a genotype and showing that each of them contributes to its resistance. This gap matters in practice, because combinations are increasingly assembled on the basis of marker data, whereas the evidence needed to judge whether a combination works, and why, is rarely made explicit. Here, we examine the factors that determine whether a marker-defined resistance gene pyramid confers effective resistance in a given genetic background and environment. Drawing on selected experimental studies, we compare evidence on gene interactions, molecular mechanisms, resistance phenotypes and agronomic performance (Figure 1). Particular attention is paid to distinguishing experimentally demonstrated effects from proposed mechanisms, and to identifying the evidence needed to predict the performance and durability of resistance gene combinations. We discuss how genetic characterization, expression analysis, causal genetic tests, functional assays and multi-environment phenotyping can together provide this evidence. We also consider how the evolution of pathogen populations and the way resistance genes are deployed affect the durability of pyramids. Finally, as genome editing and multigene constructs expand the possibilities for combining resistance alleles, we consider how their functional compatibility and performance across genetic backgrounds can be evaluated.

**Figure 2:**
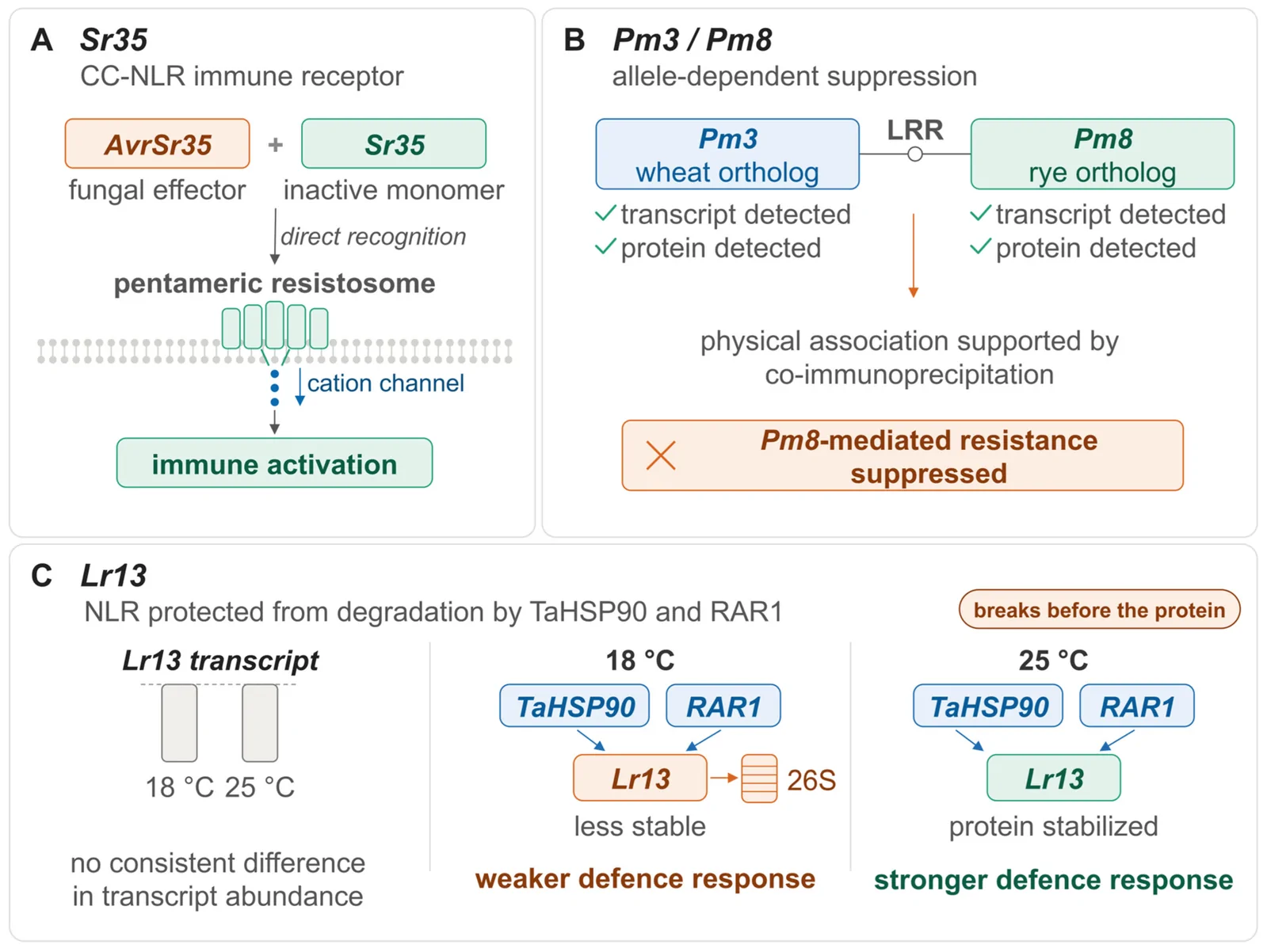
The presence of a resistance allele, and of its transcript, does not establish that the protein is functional. (**A**) *Sr35* illustrates a complete chain: direct recognition of the fungal effector *AvrSr35* triggers assembly of a pentameric resistosome with cation channel activity, linking pathogen recognition to immune activation [37]. (**B**) *Pm3* and *Pm8* are both transcribed and translated in the same plant, and co-immunoprecipitation supports their physical association through the LRR domain, yet *Pm8*-mediated resistance is suppressed. The suppression is allele-specific and independent of the recipient genetic background and was detected at the seedling stage but not in adult plants [32,33,34]. (**C**) *Lr13* transcript abundance does not differ consistently between 18 °C and 25 °C, whereas the protein is stabilized by TaHSP90 and RAR1 at 25 °C and is less stable at 18 °C, where it is degraded by the proteasome; the defence response is correspondingly stronger at the higher temperature.

**Figure 3:**
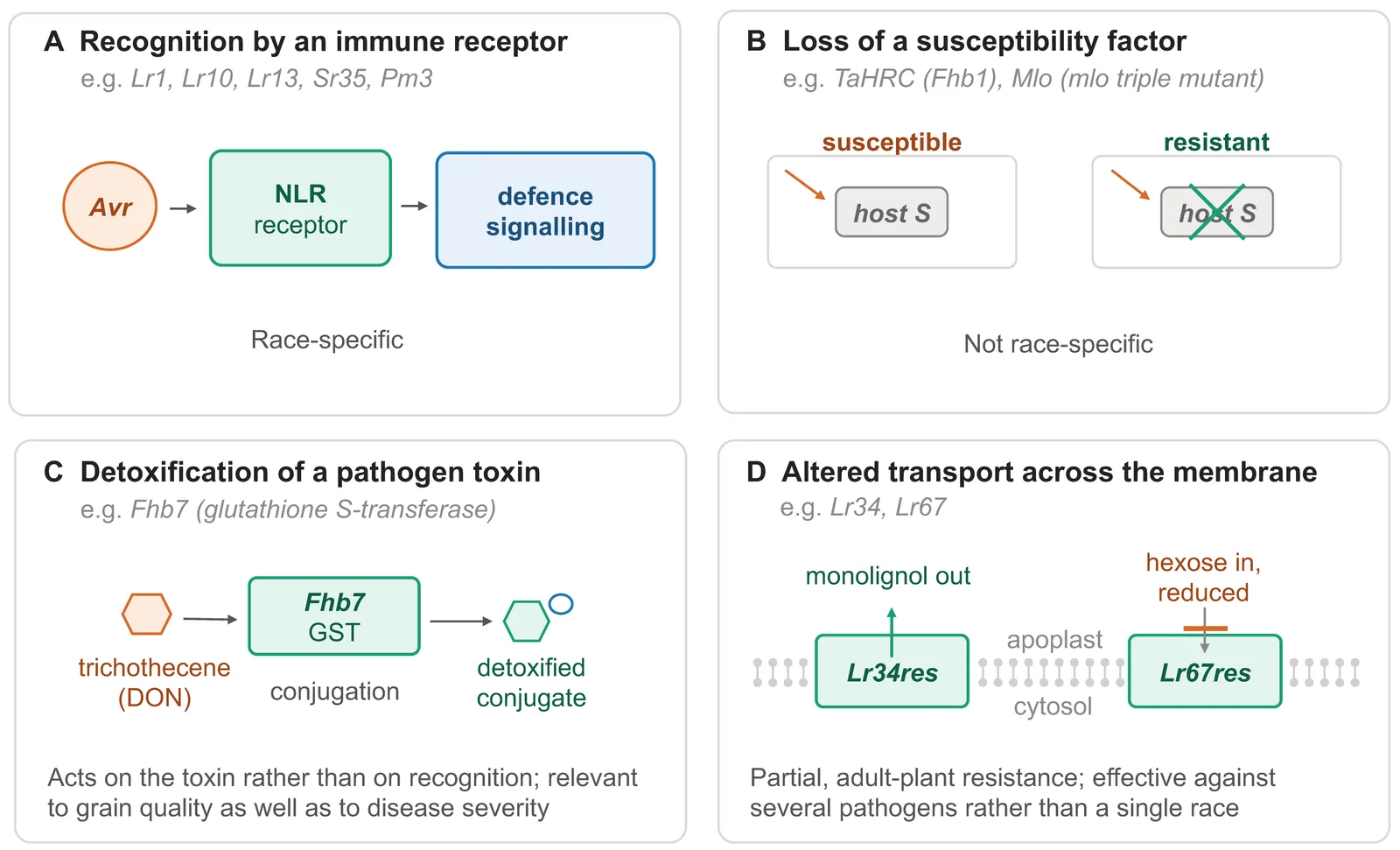
Characterized wheat resistance genes act through distinct molecular processes. (**A**) Recognition of a pathogen effector by a nucleotide-binding leucine-rich repeat (NLR) immune receptor, which oligomerizes and activates defence signalling; this mechanism is race-specific [32,33,37,38]. (**B**) Loss of a host susceptibility factor required by the pathogen, as in the *TaHRC* deletion underlying *Fhb1* [39] and in the *mlo* triple mutant [8,42]; this mechanism is not race-specific, but the host factor may have its own function, so its loss can carry a cost. (**C**) Detoxification of a pathogen toxin, as for *Fhb7*, which encodes a glutathione S-transferase that conjugates trichothecenes and is therefore relevant to grain quality as well as to disease severity [40,41]. (**D**) Altered transport across the plasma membrane, as for *Lr34* and *Lr67*, which confer partial adult-plant resistance against several pathogens [12,13,14,15,30,43].

**Figure 4:**
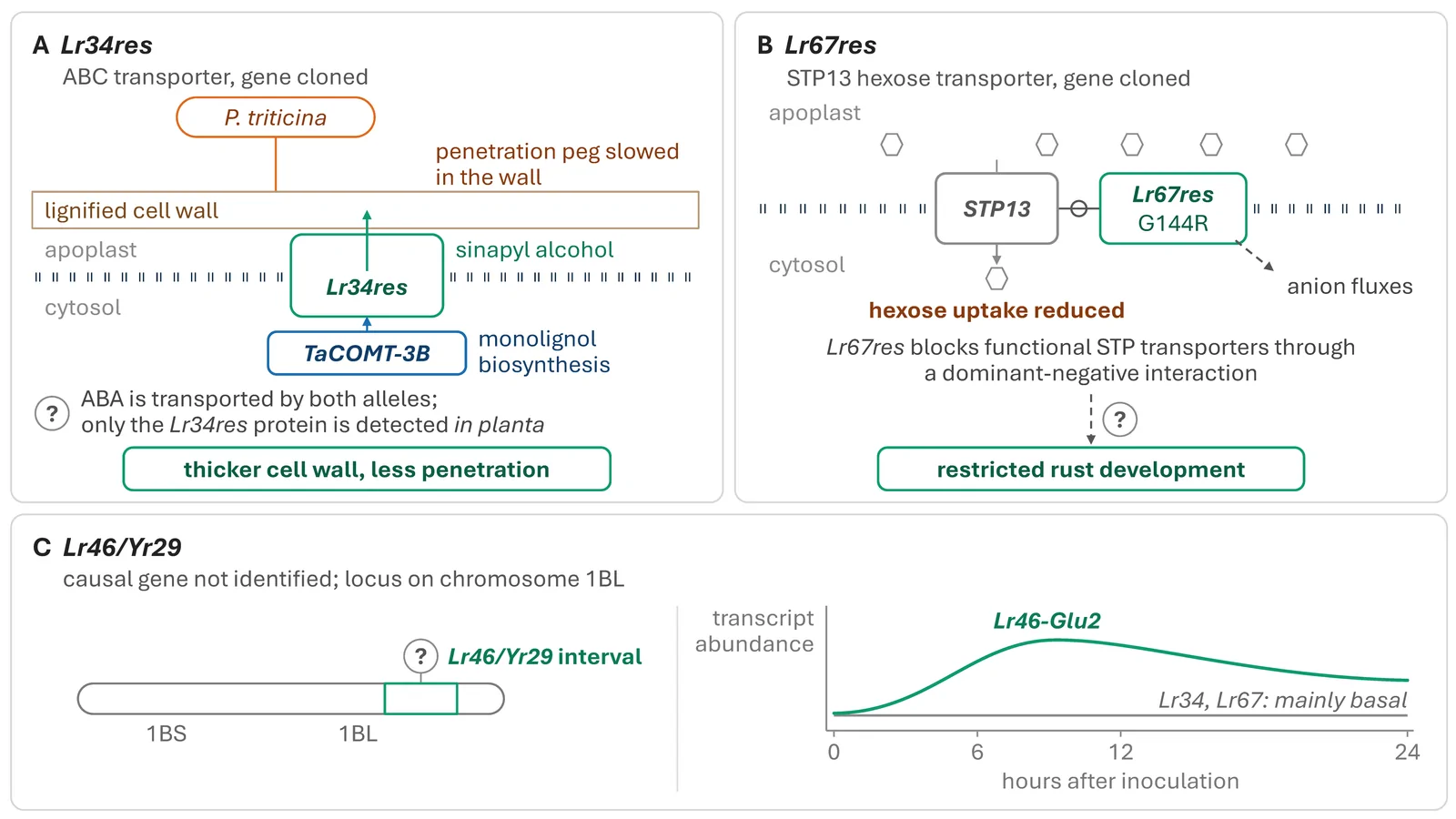
Contrasting levels of molecular characterization of the adult-plant resistance loci *Lr34*, *Lr67* and *Lr46*, shown in the context of the host cell. (**A**) The resistant allele *Lr34res* encodes an ABC transporter that exports sinapyl alcohol to the apoplast, where it contributes to cell wall lignification and slows fungal penetration; TaCOMT-3B supplies the monolignol. Both the resistant and the susceptible variants transport abscisic acid (ABA), but only the resistant protein has been detected in planta [12,13,30]. (**B**) *Lr67res* encodes a variant of the STP13 hexose transporter that reduces hexose uptake through a dominant-negative interaction with functional STP transporters; the G144R substitution alone is sufficient for resistance. Altered anion fluxes have been observed in a heterologous oocyte system, and how the altered transport restricts fungal development remains unresolved [14,15,43]. (**C**) The causal gene at the *Lr46*/*Yr29* locus on chromosome 1BL has not been identified. Of nine candidate genes examined, *Lr46-Glu2*, a putative glutamate receptor-like gene, shows an infection-associated transcriptional response, whereas *Lr34* and *Lr67* remain close to basal expression; the timing and magnitude of these responses differ among recipient backgrounds, and no consistent relationship with the associated miRNAs has been detected [9,29,44].

**Figure 5:**
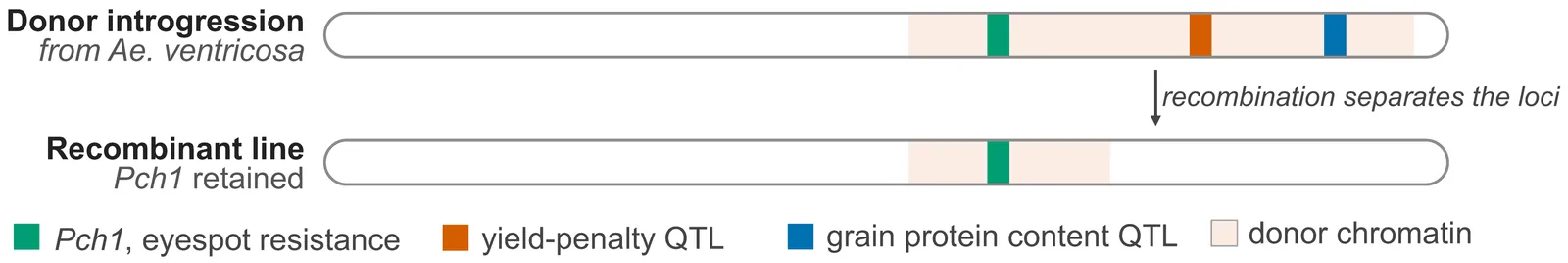
Separating a resistance gene from its genomic and physiological context. The eyespot resistance gene *Pch1* was introgressed from *Aegilops ventricosa* as part of a large 7D^V^ chromosome segment that also carries a yield-penalty QTL and a QTL for increased grain protein content. The yield penalty maps outside *Pch1* and varies between environments, whereas the effect on grain protein content is more consistent; recombination makes it possible to retain *Pch1* without the linked yield effect [19,20].

Relevant literature was identified by searching Web of Science and PubMed with combinations of terms such as “wheat”, “*Triticum aestivum*”, “gene pyramiding”, “resistance gene combination”, “gene stacking”, “adult-plant resistance”, “epistasis”, “genetic background”, “marker-assisted selection”, “linkage drag” and “genome editing”, together with the names of individual diseases and resistance genes (e.g., *Lr34*, *Lr46*, *Lr67*, *Fhb1*, *Pch1*, *Pm3* and *Sr35*). The final search was performed on 2 November 2026, and the cited publications span the years 1984–2026. The reference lists of key articles and reviews were also screened. In this review, we gave priority to experimental studies that (i) compared resistance gene combinations with their individual components in defined genetic backgrounds, (ii) provided molecular or functional evidence for the genes involved, or (iii) evaluated the agronomic consequences of introgressing or stacking resistance genes. Because this is a narrative rather than a systematic review, the selected studies are illustrative rather than exhaustive. The examples cover leaf rust, stripe rust, stem rust, powdery mildew, eyespot and Fusarium head blight (Table 1). The adult-plant resistance loci *Lr34*, *Lr46* and *Lr67* are examined in greater depth as a case study, because their contrasting levels of molecular characterization illustrate the difference between identifying a resistance gene and validating its function. All schematic figures (Figure 1, Figure 2, Figure 3, Figure 4 and Figure 5) were prepared by the authors in Microsoft PowerPoint and Microsoft Publisher (Microsoft Corporation, Redmond, WA, USA).

## 2. Interactions Between Resistance Genes and Their Phenotypic Effects

Combining multiple resistance alleles is generally expected to improve resistance, although their effects are not necessarily additive. The interpretation of such non-additive effects depends partly on how disease severity is measured and analysed. A significant statistical interaction does not establish a direct molecular relationship between the genes involved, and the absence of a significant interaction is not evidence that their effects are independent. Differences in disease pressure, genetic background and statistical power may also influence the results (Table 1).

McCallum and Hiebert [4] provide a particularly informative example. They developed six doubled-haploid populations (58–114 lines each), each segregating for either *Lr34* or *Lr67* and for one of the leaf rust resistance genes *Lr13, Lr16* or *Lr32* (Table 1). Lines carrying *Lr13* together with *Lr34* were more resistant than lines carrying *Lr34* alone. The interaction between the two genes was statistically significant, whereas no significant interaction was detected between *Lr13* and *Lr67*. Both APR genes interacted significantly with *Lr16*, while *Lr32* showed a significant interaction with *Lr67* but not with *Lr34*. Nevertheless, lines combining *Lr32* with either APR gene were more resistant than lines carrying any of these genes alone. These results show that the phenotypic benefit of combining resistance loci should be distinguished from statistical evidence of non-additivity.

The genetic composition of these populations also deserves consideration. Although most parental lines were near-isogenic, the doubled-haploid progeny could still differ at loci outside those under investigation. Furthermore, *Lr13* segregated in the *Lr32* populations but was not classified independently. The analyses also used a beta-distributed model of disease severity, so the interpretation of interaction effects depends on the scale of the model. These limitations do not invalidate the results, but they restrict conclusions about the mechanisms underlying the observed differences.

Further evidence comes from the Canadian cultivar Carberry, which has maintained a high level of leaf rust resistance despite changes in pathogen virulence. Bokore et al. [22] analysed 297 doubled-haploid lines derived from the cross Carberry × Thatcher in seven field environments. Of the nine resistance QTL detected, those corresponding to *Lr34* and *Lr46* contributed substantially to adult-plant resistance. Five epistatic interactions were identified, including favourable interactions of *Lr34* with *Lr46*, *Lr16* and *Lr2a*. In contrast, negative epistatic effects were detected between *Lr46* and *Lr16*, and between *Lr2a* and another QTL on chromosome 2B. Importantly, these negative interactions did not necessarily reduce resistance below the level conferred by the individual genes. The study illustrates that positive and negative epistatic effects can coexist within a single resistance architecture and differ considerably in their contribution to the overall phenotype.

Genetic background also influences resistance to Fusarium head blight (FHB). Brar et al. [23] examined near-isogenic lines carrying all combinations of *Fhb1*, *Fhb2* and *Fhb5* in two spring wheat backgrounds, CDC Alsask and CDC Go. Although the lines retained less than 3% of donor alleles, field resistance improved significantly in the CDC Alsask background but not in CDC Go. Several additional resistance-associated loci were identified, including favourable alleles contributed by the susceptible parent. Epistatic interactions involving markers distributed across the genome explained more than 20% of the phenotypic variation in FHB traits. These results indicate that the effects attributed to introduced resistance loci cannot always be separated from those of the recipient genome, even in advanced backcross material.

The relative contribution of the same FHB loci may also depend on which component of the infection process is assessed. Dai et al. [24] evaluated eight combinations of *Fhb1*, *Fhb2* and *Fhb5* in winter wheat doubled-haploid lines using six disease assessments. *Fhb1* was the most effective single locus after single-floret inoculation, whereas *Fhb5* performed better under natural infection. The triple combination provided the highest overall resistance. These differences are biologically relevant because initial infection and subsequent fungal spread are distinct components of FHB development. Resistance evaluated with a single inoculation method may therefore fail to capture the full contribution of a given locus.

Nevertheless, successful pyramiding across several genetic backgrounds has been demonstrated. Zhang et al. [25] introduced *Fhb1*, *Fhb4* and *Fhb5* into five modern Chinese wheat cultivars and breeding lines. Fourteen selected introgression lines showed substantially improved FHB resistance in two years of field trials, with disease severity reduced by up to 95% relative to the recipient lines. No systematic yield penalty was detected in the absence of disease. These findings show that favourable resistance combinations can remain effective across different recipient backgrounds. However, the contributions of individual loci and their possible interactions were not resolved, because a complete factorial comparison was not performed in each background.

Wang et al. [26] developed wheat lines carrying all combinations of *Yr18*, *Yr28* and *Yr36* in a common, highly susceptible background. The three loci contributed additively to stripe rust resistance in the field, and combinations of at least two genes provided substantial protection. Interestingly, lines carrying *Yr28* + *Yr36* or all three loci were also resistant at the seedling stage, even though these genes had been studied mainly for their contribution to partial resistance. Such results show that combining resistance loci can produce useful phenotypes that would not be expected from their individual effects.

Multi-environment studies raise a further consideration regarding the stability of resistance. Vo Van-Zivkovic et al. [27] analysed 35,986 lines from Australian wheat breeding populations evaluated across ten environments and identified 32 haploblocks associated with stripe rust resistance. Of these, 23 were specific to particular environments, whereas nine were associated with stable resistance across environments. Tong et al. [28] used a different panel, the Vavilov wheat diversity panel, and examined resistance in eleven field experiments in Australia and Ethiopia. They also evaluated haplotype combinations through genomic prediction and breeding simulations. Both studies demonstrate the value of considering mean resistance and its environmental stability separately. However, predicted combinations require experimental validation, particularly when the identified haploblocks do not correspond to functionally characterized resistance genes.

**Table 1:** Examples of resistance gene combinations: genetic material, test environments, interactions, resistance outcomes and agronomic effects.* n.d.—not determined in the cited study. Status summarizes the outcome reported in the cited study under the conditions tested and does not imply durability: Effective—the combination conferred higher resistance than its individual components, the recipient line or the susceptible marker class; Context-dependent—the effect of the combination differed among genetic backgrounds or environments; Suppression—one component reduced or abolished the resistance conferred by another; Unresolved—the contribution of the combination could not be determined from the available data (e.g., disease phenotype not measured, unbalanced design, or predictions not yet tested experimentally).

Combination	Material and Genetic Background	Environments and Assessment	Interaction and Resistance Outcome	Agronomic Effect	Status	Ref.
**Leaf rust (** ***Puccinia triticina*** **)**						
*Lr34* or *Lr67* with *Lr13*, *Lr16* or *Lr32*	Six DH populations (58–114 lines) from mostly near-isogenic parents	Field; all populations in the same environments	Significant interactions *Lr13* × *Lr34*, *Lr16* × *Lr34*, *Lr16* × *Lr67* and *Lr32* × *Lr67*; none for *Lr13* × *Lr67* or *Lr32* × *Lr34*. *Lr13* + *Lr34*: 17.7% severity vs. 23.8% (*Lr34*) and 84.3% (*Lr13*); *Lr32* + either APR gene more resistant than single genes	n.d. *	Effective	[4]
*Lr34*, *Lr46*, *Lr16*, *Lr2a* and other QTL (cv. Carberry)	297 DH lines, Carberry × Thatcher	Seven field environments; seedling tests	Nine QTL; synergistic epistasis of *Lr34* with *Lr46*, *Lr16* and *Lr2a*; negative epistasis (*Lr46* × *Lr16*; *Lr2a* × 2B QTL) without loss of resistance; resistance maintained despite virulence to *Lr2a*, *Lr13*, *Lr16* and *Lr23*	n.d. *	Effective	[22]
*Lr34* + *Lr46*; *Lr67* + *Lr46* (marker-defined)	Six BC_2_F_1_ combinations: two donors × three winter wheat cultivars	Controlled inoculation; 0–48 hpi	Background-dependent transcript abundance and timing; disease severity, fungal biomass and protein abundance not measured	n.d. *	Unresolved	[29]
**Stripe rust (*****Puccinia striiformis*** **f. sp.** ***tritici*****)**						
*Yr18*, *Yr28*, *Yr36* (all combinations)	BC_3_F_5_ lines in the susceptible line SY95-71	Field (adult plants); seedling tests	Additive effects; two or more genes gave adequate resistance; *Yr28* + *Yr36* and the triple combination also resistant at the seedling stage	n.d. *	Effective	[26]
*Lr34res* + favourable *TaCOMT-3B* allele	312 accessions; biparental population	Multiple field environments	Higher lignin content and lower severity than *Lr34res* alone; complementary biochemical functions (monolignol synthesis and transport)	n.d. *	Effective	[30]
Multiple resistance haplotypes (haploblocks)	35,986 Australian breeding lines; Vavilov diversity panel	10 environments; 11 field experiments in Australia and Ethiopia	32 haploblocks, nine stable across environments; in silico stacking and breeding simulations predict higher and more stable resistance	n.d. *	Unresolved	[27,28]
**Stem rust (*****Puccinia graminis*** **f. sp.** ***tritici*****)**						
*Sr22*, *Sr35*, *Sr45*, *Sr50*, *Sr55* (Big5 transgene cassette)	Single transgenic locus, cv. Fielder	Field; seedling tests with diverse isolates	Very high field resistance; at least four of five genes functional; an isolate virulent to several genes in the stack identified	n.d. *	Effective	[7]
Big5 + Fearless5 (*Sr13c*, *Sr21*, *Sr22*, *Sr26*, *Sr33*): ten transgenes, nine genes	Fielder and Robin; several independent events	Field, two seasons	High field resistance; stable inheritance and expression over eight generations	No reproducible penalty without disease	Effective	[31]
**Rusts, several species**						
Four or five APR genes	CIMMYT high-yielding spring wheat	Field, high disease pressure	Single APR genes insufficient; four or five genes usually confer near-immunity	n.d. *	Effective	[17]
**Powdery mildew (*****Blumeria graminis*** **f. sp.** ***tritici*****)**						
Pairs of *Pm3* alleles	F_1_ hybrids; stacked transgenic lines	Controlled conditions; transient assays in *Nicotiana benthamiana*	Post-translational suppression of *Pm3* resistance (RNA and protein unaffected); conferred by the LRR domain; independent of genetic background	n.d. *	Suppression	[32]
*Pm8* (1BL.1RS) + *Pm3*	1BL.1RS lines; transgenic lines; DH population	Controlled conditions; seedlings and adult plants	*Pm8* intact and expressed but suppressed; PM3 and PM8 proteins interact (co-IP); suppression at the seedling but not the adult-plant stage	n.d. *	Suppression	[33,34]
**Fusarium head blight (*****Fusarium*** **spp.)**						
*Fhb1*, *Fhb2*, *Fhb5* (all combinations)	NILs in CDC Alsask and CDC Go (<3% donor alleles)	Eight field environments; FHB and DON	Improved resistance in CDC Alsask but not CDC Go; additional loci, including alleles from the susceptible parent; epistasis explained >20% of variation	n.d. *	Context-dependent	[23]
*Fhb1*, *Fhb2*, *Fhb5* (eight combinations)	Winter wheat DH population (donor Sumai 3)	Six tests, including single-floret inoculation and natural infection	*Fhb1* most effective after single-floret inoculation, *Fhb5* under natural infection; triple combination most resistant	No yield difference between selected DH lines and recipient parents	Effective	[24]
*Fhb1*, *Fhb4*, *Fhb5*	14 introgression lines in five Chinese cultivars or lines	Field, two years, artificial inoculation	Severity reduced by up to 95% relative to recipient lines; contributions of individual loci not resolved; DON not measured	No systematic effect without inoculation	Effective	[25]
QTL on 3B + 5A (CM82036); 3A (Frontana)	Elite European spring wheat; 12–15 lines per QTL class	Four locations; spray inoculation	3B + 5A reduced DON by 78% and FHB rating by 55% relative to the susceptible class; significant differences within marker classes	n.d. *	Effective	[35]
**Eyespot (*****Oculimacula*** **spp.)**						
*Pch1* + *Q.Pch.jic-5A*	Winter wheat breeding lines (one *Pch1* line; three *Pch1* + *Q.Pch.jic-5A* lines)	Inoculation tests; yield trials	No improvement over *Pch1* alone; small, unbalanced set of lines	Lower yield with the *Ae. ventricosa* 7D^V^ segment [19]; penalty separable from *Pch1* by recombination [20]; no effect of *Pch1* on plot yield [36]	Unresolved	[19,20,36]
*Pch1* + *Pch2*	Unpublished field data presented in [1]	Field, severe disease pressure	Markedly improved eyespot control	n.d. *	Effective	[1]

## 3. Molecular Function of Resistance Genes

Molecular characterization of wheat resistance genes has revealed considerable diversity in the mechanisms of pathogen recognition and defence. Many major race-specific resistance genes, including *Lr1*, *Lr10*, *Lr13*, *Sr35* and *Pm3*, encode nucleotide-binding leucine-rich repeat (NLR) proteins, whereas others belong to structurally distinct protein families. The stem rust resistance gene *Sr35* provides one of the clearest examples of a functionally characterized immune receptor. Structural analyses showed that direct recognition of the fungal effector *AvrSr35* triggers the assembly of a pentameric resistosome with cation channel activity [37] (Figure 2A). Such studies establish a direct link between pathogen recognition and immune activation. However, understanding how an individual receptor functions does not necessarily explain how it behaves when combined with other resistance genes.

Indeed, molecular interactions between resistance proteins can interfere with their expected activity. Stirnweis et al. [32] showed that certain combinations of *Pm3* alleles suppressed powdery mildew resistance, even though both transcripts and proteins were present. The suppression was associated with the LRR domain and occurred independently of the recipient genetic background. Hurni et al. [33] described a related mechanism, in which the rye-derived resistance gene *Pm8* was suppressed by its wheat orthologue *Pm3*. Both proteins were detected in the affected plants, and co-immunoprecipitation experiments supported their physical association. More recently, Golzar et al. [34] reported that suppression of *Pm8*-mediated resistance by particular *Pm3* alleles was evident at the seedling stage but not in adult plants. These findings provide direct evidence that resistance genes may be transcribed and produce detectable proteins without conferring the expected phenotype (Figure 2B).

The importance of protein-level regulation is further illustrated by *Lr13*, an NLR gene whose resistance is temperature-sensitive. Working with wheat seedlings, Wang et al. [38] showed that TaHSP90 interacts with *Lr13* and, together with RAR1, protects it from proteasomal degradation. *Lr13* was more stable, and defence responses were stronger at 25 °C than at 18 °C, even though *Lr13* transcript abundance did not differ accordingly. Thus, within the range tested, *Lr13*-mediated resistance was enhanced at the higher temperature, and this effect was regulated at the protein rather than the transcript level (Figure 2C). Whether the same regulation operates in adult plants and outside this temperature range remains to be determined. This finding is particularly relevant because *Lr13* was one of the major resistance genes examined in the factorial experiments of McCallum and Hiebert [4], where it showed a significant interaction with *Lr34*. Although the molecular basis of that interaction remains unknown, HSP90-dependent regulation of *Lr13* offers a possible mechanism by which environmental conditions could alter the functional contribution of a resistance allele.

Not all resistance mechanisms depend on immune receptors. The *Fhb1*-associated deletion in *TaHRC* illustrates resistance resulting from the disruption of a host susceptibility factor [39], whereas *Fhb7* encodes a glutathione S-transferase implicated in resistance to Fusarium head blight and in mycotoxin detoxification [40,41]. These mechanisms differ substantially both from NLR-mediated recognition and from the transporter-associated resistance conferred by *Lr34* and *Lr67* (Figure 3). Their diversity raises an important question for gene pyramiding: are combinations of genes acting through different molecular processes more likely to provide complementary protection than combinations of genes acting through closely related pathways? This remains a hypothesis to be tested experimentally, not a general property that can be inferred from protein annotations alone.

Adult-plant resistance (APR) genes offer an opportunity to examine why the presence of a resistance allele does not always predict its phenotypic contribution. Although *Lr34, Lr46* and *Lr67* produce broadly similar slow-rusting phenotypes, the available molecular evidence suggests that they should not be treated as functionally equivalent components of resistance gene pyramids (Figure 4).

*Lr34* encodes an ABC transporter, and its resistant allele has provided durable protection against several fungal pathogens [12]. Krattinger et al. [13] showed that both the resistant and the susceptible *Lr34* variants can transport abscisic acid (ABA) in heterologous systems. However, only the resistant protein was detected in the plant material tested, which suggests that differences in protein accumulation or stability may contribute to the contrasting phenotypes. More recently, Zhang et al. [30] identified sinapyl alcohol, a monolignol involved in lignin biosynthesis, as another *Lr34* substrate. Their experiments combined transporter assays, transcriptomic and metabolomic analyses, *lr34* mutants and measurements of cell wall lignification. Wheat lines carrying *Lr34res* (the resistance-conferring allele of *Lr34*) had thicker cell walls and showed reduced fungal penetration, whereas disruption of *Lr34* was associated with impaired lignification. These findings link *Lr34* activity to structural resistance more directly than could previously be inferred from its annotation as a transporter. It remains unclear whether ABA redistribution and monolignol transport contribute to *Lr34*-mediated resistance independently or through interconnected processes (Figure 4A).

Of particular interest is the interaction between *Lr34* and *TaCOMT-3B*, a gene involved in sinapyl alcohol biosynthesis. Zhang et al. [30] examined 312 wheat accessions and a biparental population, including field evaluations of stripe rust in multiple environments. Lines carrying favourable alleles of both genes accumulated more lignin and showed lower disease severity than lines carrying *Lr34res* alone. In this example, the benefit of combining genetic factors can be interpreted in terms of complementary biochemical functions: *TaCOMT-3B* contributes to monolignol production, whereas *Lr34* facilitates its transport. Nevertheless, further research is needed to determine how much this mechanism contributes to resistance against different pathogens and whether the interaction is maintained in unrelated wheat backgrounds.

The molecular basis of *Lr67*-mediated resistance is different. *Lr67* encodes a variant of an STP13-family hexose transporter, and the resistant and susceptible alleles differ by two amino acid substitutions, G144R and V387L [14]. The resistant variant lacks conventional hexose transport activity, but recent studies indicate that its function cannot be explained solely by reduced sugar uptake. Milne et al. [15] showed that expression of *Lr67res* in *Xenopus laevis* oocytes was associated with altered anion fluxes. Subsequent experiments showed that the G144R substitution alone can confer rust resistance and that the corresponding modification of the barley STP13 orthologue increases resistance to *Puccinia hordei* [43]. These findings directly link specific changes in transporter structure to the resistance phenotype, although the role of altered ion transport in restricting fungal development remains unresolved. They also illustrate why transcript abundance alone may be an incomplete measure of resistance gene activity (Figure 4B).

The differences between *Lr34* and *Lr67* are particularly relevant when interpreting their interactions with major resistance genes. Both proteins can reduce disease severity and delay pathogen development, yet the biochemical changes associated with their resistant alleles are distinct. This offers a possible biological explanation for the different interaction patterns observed by McCallum and Hiebert [4]. However, the available evidence does not demonstrate a causal relationship between transporter function and the statistical interactions detected in their field populations. Establishing such a relationship would require comparing the relevant allele combinations in more closely controlled genetic backgrounds, together with measurements of transporter activity and pathogen development.

*Lr46/Yr29* presents a different problem, because the causal gene at this locus has not been identified [9]. Studies of the chromosome 1BL region have identified several candidate genes, but it remains uncertain whether the broad-spectrum resistance associated with this locus results from a single pleiotropic gene or from several closely linked genes. Spychała et al. [44] examined the expression of nine candidate genes and four associated miRNAs following *Puccinia triticina* infection. Among the candidates, *Lr46-Glu2*, which encodes a putative glutamate receptor-like protein, showed an infection-associated transcriptional response that warrants further investigation. However, neither its temporal expression pattern nor its association with linked molecular markers establishes that *Lr46-Glu2* is responsible for the resistance phenotype. Fine mapping, targeted mutagenesis and complementation experiments will be needed to confirm its function (Figure 4C).

Expression studies in reference cultivars and successive hybrid generations have added another dimension to the analysis of APR loci. Following *P. triticina* inoculation, differences have been reported in the abundance and temporal profiles of transcripts of *Lr34*, *Lr67* and *Lr46*-associated genes [44,45,46,47]. More recently, Spychała et al. [29] examined six BC_2_F_1_ wheat combinations derived from crosses between each of two donors, TX89D6435 and Purdue, and three recipient elite cultivars: Euforia, Medalistka and Symetria. RT-qPCR analysis revealed substantial variation among genotypes in transcript abundance and in the timing of responses. *Lr34* and *Lr67* showed mainly basal expression, without consistent induction after infection, whereas the candidate gene *Lr46-Glu2* tended to respond earlier, particularly 6–12 h after inoculation. However, the magnitude and timing of these responses differed among recipient backgrounds. The accompanying ddPCR analyses also revealed genotype- and time-dependent variation in miRNA abundance, but this did not consistently correspond to the abundance of the putative target transcripts. No significant overall correlation was detected between the expression of tae-miR5384-3p and *Lr46-Glu2*. These observations do not exclude post-transcriptional regulation, but they provide no direct evidence of a consistent miRNA-mediated regulatory relationship under the conditions examined. An important limitation is that the BC_2_F_1_ materials carried the marker-defined combinations *Lr34* + *Lr46* or *Lr67* + *Lr46*, but not all three APR loci. Moreover, the study characterized transcriptional responses without quantifying disease severity, pathogen biomass or protein abundance. Although expression profiles differed between genetic backgrounds, these differences cannot yet be attributed to specific regulatory mechanisms or used to predict resistance. This distinction is particularly important for *Lr34* and *Lr67*, for which changes in protein function and accumulation may be more informative than transcript levels alone.

## 4. Agronomic Consequences of Gene Pyramiding

The effectiveness of a resistance gene combination is only one aspect of its breeding value. Resistance alleles are often introduced from wild relatives or genetically distant donors, and the chromosomal segments that carry them may also affect yield, grain quality or other agronomic traits. The performance of an introgression line therefore cannot always be attributed to the resistance genes tracked with molecular markers. Even in the absence of linkage drag, a resistance gene may interact epistatically with loci that determine agronomic performance, so that its effect depends on the genetic background into which it is introduced. In other cases, the cost is inherent to the resistance mechanism itself, because the gene product that contributes to disease resistance also has undesirable effects on quality or end use. Lignin deposition, for instance, reinforces cell walls against pathogen ingress but reduces forage digestibility, and glucosinolates contribute to the defence of oilseed rape while acting as antinutritional compounds in rapeseed meal. Linkage drag can be reduced by recombination, and background effects by the choice of recipient germplasm, but pleiotropic costs cannot be separated from the resistance they accompany. Distinguishing among these sources is therefore essential when the costs of resistance are assessed, because only pleiotropic costs are attributable to resistance as such.

Two sources of such costs should be distinguished (Figure 5). Linkage drag arises from donor chromatin that is inherited together with the resistance gene; because it is caused by neighbouring genes, it can in principle be removed by recombination or avoided altogether by transferring the cloned gene alone. Pleiotropic effects arise from the resistance gene itself or from the way it is expressed; they persist regardless of the size of the donor segment, but may depend on expression level, allele type or genetic background. Both are discussed below, because they call for different breeding solutions.

Kwiatek et al. [19] compared winter wheat breeding lines carrying *Pch1*, *Pch2*, *Q.Pch.jic-5A* or a combination of *Pch1* and *Q.Pch.jic-5A*. The combination showed no significant improvement in resistance over *Pch1* alone, and lines carrying the *Pch1*-associated *Aegilops ventricosa* introgression had lower grain yield. However, the set of lines was small and unbalanced: only one genotype carried *Pch1* alone, and three carried *Pch1* together with *Q.Pch.jic-5A*. With these data, it was not possible to separate the effect of the resistance locus from that of linked donor chromatin or from other differences between the breeding lines. By contrast, unpublished field data presented by Mundt [1] showed that combining *Pch1* with *Pch2* markedly improved eyespot control under severe disease pressure, suggesting that the benefit of a combination depends on the partner locus as much as on *Pch1* itself. Later studies gave a broader picture. Majka et al. [36] confirmed that *Pch1* was the main source of eyespot resistance in winter wheat breeding material but found no significant effect of *Pch1* or *Pch2* on plot yield. Lines carrying *Pch1* even had a higher thousand-kernel weight than lines carrying only *Pch2* or neither gene. Wiśniewska et al. [48] then showed that marker-assisted selection works well during doubled-haploid production: the 65 *Pch1*-positive lines they obtained were more resistant to eyespot than the 30 lines without the gene. Neither study explained the genetic basis of the yield reduction reported earlier, but both showed that *Pch1* was not consistently associated with poor agronomic performance. More direct evidence came from Pasquariello et al. [20], who analysed recombinant lines derived from the *Ae. ventricosa* 7D^V^ introgression. They mapped a yield-penalty QTL and a QTL for increased grain protein content to a region genetically distinct from *Pch1*. The yield effect varied between environments, whereas the effect on grain protein content was more consistent. Recombination also made it possible to separate *Pch1* from the marker associated with reduced yield (Figure 5). These results suggest that at least part of the yield penalty historically attributed to *Pch1* was caused by the accompanying donor segment rather than by the resistance gene itself. They also show how valuable recombination and background selection are when resistance is transferred from wild relatives.

Resistance to FHB shows that pyramiding can improve disease control without necessarily reducing agronomic performance. Zhang et al. [25] used marker-assisted backcrossing to introduce *Fhb1*, *Fhb4* and *Fhb5* into five modern Chinese wheat backgrounds. Over two years of field testing, the 14 selected introgression lines were substantially more resistant than the corresponding recipient lines, with disease severity reduced by up to 95%. In the absence of inoculation, this improvement was not accompanied by a systematic reduction in yield or in the other agronomic traits measured. Individual lines nevertheless differed in plant height, grain weight and other traits, which shows that recovering the recipient phenotype remains an important part of the breeding process. For FHB, however, disease severity alone may not be enough to establish the value of a resistance combination. Mycotoxin contamination of the grain is another important breeding target, and better visual resistance does not automatically mean lower toxin levels. Zhang et al. [25] expected their introgression lines to accumulate less deoxynivalenol (DON), but did not measure it. Earlier work by Miedaner et al. [35] shows the value of a more comprehensive evaluation. In elite European spring wheat carrying QTL from non-adapted donors, combining the resistance loci on chromosomes 3B and 5A reduced DON content by 78% and FHB severity by 55% compared with the susceptible QTL class. Genotypes within the same marker class still differed significantly, so the authors recommended using phenotypic selection alongside molecular markers. This conclusion still applies to modern pyramiding: markers can efficiently assemble favourable loci, but the breeding value of a combination must ultimately be judged on the traits it is meant to improve.

Cloned resistance genes offer a way around some of the limitations of conventional introgression. Luo et al. [7] developed a five-transgene cassette containing four major stem rust resistance genes (*Sr22*, *Sr35*, *Sr45* and *Sr50*) and the pleiotropic APR gene *Sr55/Lr67*. At least four of the five genes were functional, and the transgenic lines were highly resistant to several virulent isolates of *Puccinia graminis* f. sp. *tritici*. However, the authors also detected an isolate virulent to several genes in the stack, which underlines the need to distinguish broad resistance at the time of testing from long-term durability. In a follow-up study, Luo et al. [31] combined the Big5 and Fearless5 transgene loci to produce wheat plants carrying ten resistance transgenes. These represented nine distinct genes, because *Sr22* was present in both constructs. The transgenes were stably inherited and expressed over eight generations, and the lines remained highly resistant in field trials. Comparisons across two wheat backgrounds (Fielder and Robin) and several independent insertion events revealed no reproducible agronomic penalty over two field seasons without disease pressure. These results contrast with reports of reduced yield in lines carrying some conventionally introgressed *Sr* genes, and they suggest that linked donor chromatin, rather than the resistance genes themselves, may explain at least some of those earlier observations. Introducing cloned genes without large donor chromosome segments may therefore overcome an important limitation of conventional resistance breeding. Transgene stacking does not, however, remove the need to evaluate gene expression, inheritance, disease resistance and agronomic performance in each recipient background. The absence of a detectable yield penalty under the conditions tested does not guarantee that every combination will behave the same way in other cultivars or environments. Nor should strong resistance to the isolates used in experimental trials be confused with proven durability under long-term field deployment. A cost caused by the resistance gene itself is not removed by delivering the gene without donor chromatin, but it may still depend on how the gene is expressed [8,18,42].

## 5. Pathogen Evolution and the Durability of Resistance Gene Pyramids

The rationale for pyramiding rests on pathogen evolution, so the value of a resistance gene combination cannot be judged without considering the pathogen populations it will face. The risk that a pathogen overcomes resistance increases with its population size, mutation rate, capacity for gene flow and frequency of sexual recombination [2,49]. Resistance genes grown over large areas therefore exert strong and widespread selection for virulence, and the way they are deployed influences how quickly that virulence accumulates [50].

Stem rust shows how quickly race-specific resistance can be overcome when it depends on single genes. The race Ug99, first detected in Uganda in 1998–1999, was virulent to *Sr31*, a gene present in wheat cultivars grown on a very large area, and variants of the same lineage subsequently acquired virulence to *Sr24* and *Sr36*, which had been used in their place [51,52]. These races spread through eastern and southern Africa and into the Middle East. Two breeding responses followed: (i) combining several effective race-specific genes, including stacks of cloned genes introduced at a single locus [7,31], and (ii) accumulating four or five minor-effect adult-plant resistance genes, which together provide near-immunity while each exerts little selection pressure on the pathogen [17,52].

Stripe rust provides a second example. Since 2011, populations of *Puccinia striiformis* f. sp. *tritici* in Europe have been largely replaced by new races originating from sexually recombining populations in the near-Himalayan region, which differ markedly in virulence from the races they displaced [53]. Migration of this kind means that the pathogen population a pyramid faces can change abruptly and independently of local selection. Race-specific *Yr* genes have repeatedly been overcome, whereas high-temperature adult-plant resistance, which is expressed as plants mature and temperatures rise, has remained effective for longer [54]. Combinations of adult-plant resistance genes such as *Yr18*, *Yr28* and *Yr36* can provide substantial field protection [26], but their long-term effectiveness against the new populations has not been tested.

In powdery mildew, the main lesson for pyramiding has come from interactions between resistance proteins rather than from virulence evolution: some *Pm3* alleles suppress the resistance conferred by other *Pm3* alleles or by *Pm8* [32,33,34], so combining race-specific *Pm* genes does not guarantee additive protection. Loss-of-function *mlo* resistance, by contrast, does not depend on recognition of a particular effector [8,42]. Across these diseases, therefore, the expected benefit of a pyramid depends both on the compatibility of its components and on the virulence present in the pathogen population.

How resistance genes are deployed matters as much as which genes are combined. A pyramid is more likely to remain effective when its component genes are not also deployed singly in other cultivars grown in the same region, because single-gene deployment allows the pathogen to accumulate virulence step by step [1,3]. Diversifying resistance in space and time, for example through cultivar mixtures, regional deployment or rotation of resistance sources, reduces the selection pressure imposed by any single gene [50]. Virulence frequencies also differ between regions, so a combination that is effective in one region may already be compromised in another. Surveillance of pathogen populations is therefore an essential complement to pyramiding, because it reveals which components of a combination are already ineffective before the combination is released [52,53].

Finally, does the type of resistance mechanism affect durability? In general, quantitative and adult-plant resistance has remained effective for longer than race-specific resistance, although erosion of quantitative resistance has also been documented [55,56]. *Lr34*, for example, has remained effective for more than 50 years of widespread use [12]. It is often argued that combining genes that act through different molecular processes (Figure 3) should make a pyramid harder to overcome, because the pathogen would require several independent adaptations. This expectation is consistent with evolutionary reasoning [56], but we are not aware of experimental studies that have compared the durability of combinations that differ in their molecular mechanisms. Because durability can only be established retrospectively [11], the hypothesis will require long-term monitoring of deployed combinations, together with information on which of their components the pathogen has overcome.

## 6. Beyond Marker-Assisted Selection

Evaluating resistance gene combinations requires several types of evidence, each addressing a different aspect of their genetic, molecular or phenotypic performance (Table 2). Gene expression analysis is one of these approaches: it can show how resistance-associated loci respond to infection in different genetic backgrounds, but it cannot by itself demonstrate protein activity or a causal contribution to resistance. Reverse-transcription quantitative PCR (RT-qPCR) is a relatively accessible method for comparing transcript abundance before and after inoculation, at different stages of infection and, where relevant, at different developmental stages. Such analyses are relevant to APR genes, whose expression may differ between genetic backgrounds even without strong induction after infection [29]. A further complication is that the resistant and susceptible alleles of *Lr34* differ by only a few polymorphisms [12], and both *Lr34* and *Lr67* have susceptible alleles or homoeologous copies that are expressed in most wheat genotypes [14,57]. RT-qPCR assays should therefore be allele- and homoeologue-specific; otherwise, measured transcript levels may reflect the combined expression of resistant and susceptible copies rather than that of the resistance allele itself. In our recent study, the transcriptional behaviour of APR-associated genes differed considerably among wheat genotypes carrying the same resistance loci [29].

These types of evidence differ in what they can establish. Table 2 summarizes this hierarchy of validation evidence, whereas Figure 2 provides biological examples of why detecting a resistance allele or its transcript does not establish functional resistance. Marker confirmation shows that an allele, or a sequence linked to it, is present. Expression evidence shows that a gene or candidate gene is transcribed and how its transcript abundance changes after infection. Causal genetic validation, through mutagenesis, gene silencing, transgenic complementation or targeted editing, shows that the gene is required for or sufficient to confer the resistance phenotype. Molecular function shows what the protein does, for example in transport, enzyme or protein-interaction assays. Field-level validation shows whether the resistance, and the combination of which the gene is part, is effective and agronomically acceptable across environments. Each level answers a different question, and a higher level does not make a lower one redundant: a gene validated by complementation may still fail to act in a particular combination or environment (Figure 2B,C), and a combination that performs well in the field may include components that contribute little. In this review, we regard a resistance gene as functionally validated when causal genetic evidence is available, and a combination as validated when its components have been compared, individually and together, in defined genetic backgrounds and in more than one environment.

How such data are interpreted depends largely on the experimental design. Near-isogenic lines and well-designed segregating populations allow more informative comparisons than unrelated cultivars that differ in many resistance and agronomic traits. Comparing lines carrying individual resistance genes with lines carrying their combinations, ideally in a common genetic background, would help establish whether changes in expression are associated with the presence of additional loci. Allele dosage should also be considered. Plants from early backcross generations are heterozygous at the introduced loci, and homozygous lines derived from them would allow dosage effects to be separated from background effects [29]. Ideally, transcript abundance and disease development should be measured in the same plant material. Pathogen biomass can be quantified by qPCR in the same tissue samples, which provides a direct link between expression data and fungal development, including at early time points when symptoms are not yet visible.

Sampling time, tissue and developmental stage all need careful attention, as does reference-gene stability, which may change during infection; RT-qPCR analyses should therefore follow the MIQE guidelines [58]. Even so, differences in transcript abundance alone cannot show that resistance genes interact through a regulatory mechanism. Such conclusions require additional evidence, for example from protein-level analyses, targeted genetic manipulation or complementation experiments. Similarly, well-controlled phenotypic comparisons do not necessarily reveal the molecular basis of an interaction, and transcriptional differences between genetic backgrounds should not automatically be taken as evidence of epistasis.

The value of a resistance gene combination also has to be judged in the context of breeding. Multi-environment field trials remain essential to establish whether resistance is sufficiently stable and whether it comes with undesirable agronomic effects. Long-term durability is a further challenge, because resistance that holds over a few experimental seasons does not necessarily predict how it will perform against evolving pathogen populations (Section 5).

Not all of these approaches are suited to routine breeding (Table 2). Gene-specific markers and genotyping arrays can be applied to thousands of lines at a low cost per data point and are therefore suited to early-generation selection [59,60]. Genomic prediction can capture the contribution of many small-effect loci, including those of the genetic background, and has achieved useful accuracies for adult-plant rust resistance in breeding populations [61]. Rapid generation advance, such as speed breeding, shortens the time needed to fix and evaluate new combinations [62]. By contrast, allele-specific RT-qPCR, quantification of pathogen biomass, mutagenesis, complementation and protein-level assays are slower and more expensive and require controlled inoculation; they are best reserved for parental lines, for a small number of candidate combinations, or for explaining why a combination behaves unexpectedly. A practical scheme therefore uses inexpensive, high-throughput methods to assemble and screen combinations, and applies functional and multi-environment evaluation to the few that are advanced towards release.

The rapid development of genome editing may encourage the view that combining resistance genes is now mainly a technical problem. However, the ability to introduce or modify multiple resistance alleles does not remove the biological complexity of their interactions. Even precisely edited genes remain subject to the effects of genetic background, regulatory mechanisms and environment. Editing of the three *Mlo* homoeologues illustrates this point. Loss of *Mlo* function conferred broad-spectrum resistance to powdery mildew [8] but is often associated with growth penalties, whereas a specific deletion allele that also activated a neighbouring sugar transporter gene provided resistance without reducing growth or yield [42]. Greater precision in genome modification does not, therefore, necessarily lead to more predictable resistance.

Genome editing opens specific possibilities for combining resistance mechanisms. Multiplex CRISPR/Cas9 editing can target several genes, together with their homoeologues, in a single transformation, and Cas9 activity that persists across generations can generate additional mutations in the progeny [63]. Susceptibility genes, whose loss of function confers resistance, are obvious targets [8,42]. Resistance alleles that differ from susceptible ones by only a few amino acids, such as *Lr67res*, in which the G144R substitution alone confers resistance [43], could in principle be recreated by precise editing in elite cultivars without linkage drag. The growing number of cloned resistance genes, accelerated by approaches that combine sequence capture with association genetics [64], also increases the supply of genes that can be stacked as single-locus cassettes [7,31]. Several limitations remain. Loss-of-function edits usually have to be made in all three homoeologues, and incomplete knockout may leave residual susceptibility; such edits can carry agronomic costs, as shown for *mlo* [42]; transformation of wheat remains strongly genotype-dependent; and the regulation of edited and transgenic lines differs between countries. Above all, edited combinations require the same validation as conventional ones: confirmation of the edits, evaluation of the resistance conferred by each component and by the combination, and multi-environment testing of resistance and agronomic performance.

The practical challenge, therefore, is not to replace marker-assisted selection with increasingly complex molecular assays, but to use complementary genetic, molecular and phenotypic evidence to decide which combinations of resistance genes are worth keeping in breeding programmes. Markers for known resistance genes could be complemented by genomic prediction models that account for genotype-by-environment interactions [27,28] and capture the contribution of the wider genetic background. For APR loci in particular, expression studies will be most informative when they are combined with causal genetic tests, protein-level analyses and measurements of pathogen biomass in well-designed genetic comparisons, which could help explain why apparently similar gene combinations perform differently in different wheat backgrounds.

## 7. Conclusions and Perspectives

Combining resistance genes remains one of the most effective ways to improve disease resistance in wheat, but the presence of several resistance alleles does not by itself predict how well, or for how long, a combination will protect the crop. The studies reviewed here cover leaf rust, stripe rust, stem rust, powdery mildew, eyespot and Fusarium head blight, and they range from marker surveys and factorial field experiments to structural studies of immune receptors, transporter assays and multi-environment yield trials. Across this range, it can be concluded that the performance of a combination is not a property of its component genes alone, but of those genes in a particular genome, in a particular environment and against a particular pathogen population, and it therefore has to be demonstrated rather than inferred from a marker profile.

For breeding practice, these conclusions translate into a sequence of decisions rather than a single rule. The choice of components should start from the virulence present in the target region, because surveillance data show which genes are already matched and therefore add nothing to a new combination [52,53]; where possible, combinations of genes acting through different molecular processes may be prioritized for experimental evaluation, although their greater durability has yet to be demonstrated (Section 5), and allele pairs known to suppress one another should be avoided [32,33,34]. At the selection stage, markers remain the method of choice: gene-specific assays can be applied to thousands of lines at a low cost per data point [59,60], genomic prediction can additionally capture small-effect loci and genotype-by-environment interaction [27,28,61], and rapid generation advance shortens the time needed to fix a combination [62]. Two precautions are important here. The relationship between a marker and its locus has to be verified, because markers that are only linked to a locus may give discordant results [9], and background genotyping should be used from the earliest generations to select recombinants that retain the resistance gene without the surrounding donor segment [20,25].

Evaluation then has to match the trait that is to be improved. Lines carrying each component and the combination should be compared in a common background and in more than one environment, because the ranking of loci can change with the method of assessment: *Fhb1* was the most effective single locus after single-floret inoculation, whereas *Fhb5* performed better under natural infection [24]. For FHB, mycotoxin content should be measured alongside visual severity, since the two do not always respond in parallel [25,35]; for the rusts, both seedling and adult-plant tests are informative, because combinations of APR genes can confer all-stage resistance [26]. Yield and grain quality should be assessed in the target environments both with and without disease, so that pleiotropic effects and linkage drag can be distinguished from the value of the resistance itself [19,20,31,36]. Slower methods, such as allele- and homoeologue-specific RT-qPCR, protein-level assays, quantification of pathogen biomass and causal genetic tests, are best reserved for parental lines, for the few combinations advanced towards release, and for explaining why a combination behaves unexpectedly (Table 2). Release is not the end of the decision: a combination retains its advantage only if its components are not deployed singly in other cultivars grown in the same region, and if resistance sources are diversified in space and time [1,3,50].

The economics of this sequence are changing, and this affects where the decisive selection is made. Mid-density targeted genotyping panels that combine genome-wide SNPs with trait-specific markers now cost on the order of ten US dollars per sample, so whole early generations, rather than selected subsets, can be screened [65]. The step that gains most is negative selection, because this is where the cost of a breeding programme is actually saved: lines that lack the target alleles, that carry components already matched by prevalent virulence, or that still retain donor segments associated with yield or quality penalties can be discarded before they enter multi-environment trials, mycotoxin analysis or any of the slower functional assays. Negative selection does not identify the best combination, but it reduces the population to a size at which the more informative and more expensive evidence described above can realistically be collected, and it is therefore the point at which inexpensive markers contribute most to the value of the programme.

Integrating the resulting data is then the main bottleneck, and this is where machine learning is most likely to help. Marker genotypes, expression profiles, multi-environment disease and agronomic scores, high-throughput phenotyping data and virulence surveys are generated on different scales and at different times, and the relationships between them (for example, epistasis between resistance loci, genotype-by-environment interaction and the dependence of a gene’s effect on the genetic background) are precisely the non-linear relationships that conventional linear prediction models capture poorly [66]. Machine learning and neural network approaches are being developed for this purpose, both for predicting disease resistance from genomic data [66] and for combining genomic with phenomic information in wheat [67]. However, there are two important limitations. Such models describe patterns in the data used to train them, so their usefulness depends on training sets that represent the backgrounds, environments and pathogen populations of interest; and a model that predicts a resistance phenotype accurately still does not establish which component of a combination is responsible for it. Prediction and causal validation are therefore complementary rather than alternative, and the levels of evidence set out in Table 2 remain necessary even when predictive accuracy is high.

Several knowledge gaps limit how far these recommendations can currently be taken. For a number of resistance loci that are already widely used in breeding, the causal gene has not been identified, which precludes markers diagnostic of the gene itself rather than of linked sequence, and rules out recreating the resistance allele by precise editing. Factorial comparisons of components and combinations in common genetic backgrounds are rare outside a few model sets [4,24,26], and few studies have measured transcript abundance, protein accumulation and pathogen development in the same plant material [29]. How transporter-type APR proteins restrict fungal development is only partly resolved [15,43]. Whether suppressive interaction between resistance proteins is more widespread among the genes that breeders actually combine has not been examined systematically. Above all, long-term field data on defined combinations, linked to virulence surveys in the pathogen populations they face, are largely lacking, so durability continues to be assessed retrospectively [11].

We therefore see five priorities: (1) completing the molecular characterization of widely deployed resistance loci; (2) evaluating combinations in near-isogenic or factorial designs across environments, with allele-specific expression analysis, protein-level assays and pathogen biomass measured in the same material; (3) testing whether combinations of genes acting through different molecular processes are more durable than combinations acting through the same process, and whether suppressive interactions are more common than is currently recognized; (4) integrating marker data, genomic prediction and pathogen surveillance, increasingly through machine-learning models able to accommodate epistasis and genotype-by-environment interaction, so that combinations can be chosen with knowledge of both their expected performance and the virulence they will encounter [66,67]; and (5) reporting agronomic performance, together with resistance data, so that the breeding value of published combinations can be judged.

For practice, the division of labour is clear: markers should be used to assemble combinations efficiently and cheaply, whereas the decision to release a combination should rest on phenotypic, agronomic and surveillance evidence obtained in the target environments. As genome editing and single-locus gene cassettes widen the range of alleles that can be combined, this distinction becomes more rather than less important, because the technical ability to assemble a combination still says nothing about how well, or for how long, it will perform in the field. Cheaper genotyping and better predictive models will allow far more combinations to be considered than can ever be tested in the field; deciding which of them deserve testing is the task that this framework of evidence is meant to serve.

## Figures and Tables

**Table 2:** Approaches to evaluating resistance gene combinations in wheat, their principal uses and main limitations.

Level	Methods	Principal Use and Scale	Main Limitation	Examples
Genetic	PCR and KASP markers, allele sequencing, haplotype analysis, background genotyping	Assembling and tracking combinations; high throughput and low cost per sample, suited to early-generation selection	Shows presence, not function; linked markers may not tag the causal polymorphism	*Lr34* [12]
Transcriptional	RT-qPCR, RNA-seq, miRNA quantification (ddPCR)	Characterizing responses of genes and candidates to infection; research scale, controlled inoculation	Transcript levels do not reflect protein abundance or activity; non-specific assays also detect susceptible copies	*Lr13* [38]; *Lr34*, *Lr67* [29]
Molecular and functional	Mutagenesis, silencing, complementation; transport and enzyme assays; protein interaction studies; metabolite analysis	Establishing causality and mechanism; research scale, for selected genes or parental lines	Activity of a single gene does not predict its effect in combination	*Pm3*, *Pm8* [32,33,34]; *Lr34* [13,30]; *Lr67* [15,43]
Phenotypic	NILs and factorial populations; inoculation with characterized isolates; disease severity and progression; fungal biomass (qPCR)	Quantifying the contribution of each component and of their interaction; experimental populations	Statistical interaction depends on scale and power and does not imply molecular interaction	*Lr34*, *Lr67* [4]; *Fhb1*, *Fhb2*, *Fhb5* [23,24]
Agronomic	Multi-location, multi-year trials; yield, grain quality and DON; G × E analysis	Decisions on release; breeding scale, in the target environments	Valid only for tested environments; durability can only be judged retrospectively; yield penalties may arise from linked donor chromatin	*Pch1* [19,20]; transgene stacks [7,31]; *Fhb1*, *Fhb4*, *Fhb5* [25]

## Data Availability

No new data were created or analyzed in this study. Data sharing is not applicable to this article.

## Abbreviations

The following abbreviations are used in this manuscript:

ABA	abscisic acid
ABC	ATP-binding cassette
APR	adult-plant resistance
Cas9	CRISPR-associated protein 9
CRISPR	clustered regularly interspaced short palindromic repeats
ddPCR	droplet digital PCR
DON	deoxynivalenol
*Fhb*	Fusarium head blight resistance gene/QTL
FHB	Fusarium head blight
G × E	genotype-by-environment interaction
HSP90	heat shock protein 90
KASP	Kompetitive allele-specific PCR
*Lr*	leaf rust resistance gene
LRR	leucine-rich repeat
MIQE	Minimum Information for Publication of Quantitative Real-Time PCR Experiments
miRNA	microRNA
*Mlo*	Mildew Resistance Locus O
NIL	near-isogenic line
NLR	nucleotide-binding leucine-rich repeat receptor
PCR	polymerase chain reaction
*Pch*	eyespot resistance gene
*Pm*	powdery mildew resistance gene
qPCR	quantitative PCR
QTL	quantitative trait locus/loci
R gene	race-specific (major) resistance gene
RNA-seq	RNA sequencing
RT-qPCR	reverse-transcription quantitative PCR
*Sr*	stem rust resistance gene
STP	sugar transport protein
*Yr*	yellow (stripe) rust resistance gene
